# Comparative assessment of Riesling wine fault development by the electronic tongue and a sensory panel

**DOI:** 10.1111/1750-3841.17036

**Published:** 2024-03-27

**Authors:** Rachel I. Potter, Claire A. Warren, Jungmin Lee, Carolyn F. Ross

**Affiliations:** ^1^ School of Food Science Washington State University Pullman Washington USA; ^2^ Horticultural Crops Production and Genetic Improvement Research Unit United States Department of Agriculture (USDA) Agricultural Research Service (ARS) Corvallis Oregon USA

**Keywords:** flaws, nondestructive, rate‐all‐that‐apply, white wine

## Abstract

**Abstract:**

Wine faults threaten brand recognition and consumer brand loyalty. The objective of this study was to compare the acuteness of e‐tongue and human sensory evaluation of wine fault development in Riesling wine over 42 days of storage. Riesling wines uninoculated (control) or inoculated with 10^4^ CFU/mL cultures of *Wickerhamomyces anomalus*, *Acetobacter aceti*, *Lactobacillus brevis*, or *Pediococcus parvulus* were assessed every 7 days with the e‐tongue and a rate‐all‐that‐apply (RATA) sensory panel. After 7 days of storage, the e‐tongue detected differences in all four wine spoilage microorganism treatments, compared to control wine, with discrimination indices over 86%. The RATA sensory panel detected significant differences beginning on day 35 of storage, 28 days after the e‐tongue detected differences. This study showed that the e‐tongue was more sensitive than the human panel as a detection tool, without sensory fatigue.

**Practical Application:**

This research is useful for winemakers seeking additional instrumental methods in the early detection of wine faults. Given the results of this study, the e‐tongue can be a useful tool for detecting early chemical changes in white wines that have undergone microbial spoilage, providing winemakers with time to mitigate faults before they surpass sensory thresholds.

## 1 INTRODUCTION

Wine fault monitoring is important in wine production to mitigate faults prior to releasing wines to consumers (Grainger, 2021). The genera *Wickerhamomyces*, *Acetobacter*, *Lactobacillus*, and *Pediococcus* are some key microbiological spoilage organisms that can affect the wine chemically. *Wickerhamomyces anomalus* is a strong producer of acetate esters including ethyl acetate and isoamyl acetate, associated with the aromas of nail polish remover and banana, respectively (Padilla et al., 2018). In Muscat fermentations, previous work showed that *W. anomalus* produced high concentrations of ethyl acetate and isoamyl acetate compounds (Padilla et al., 2018; Viana et al., 2008). *Acetobacter aceti* is associated with high‐volatile acidity in wines (Drysdale & Fleet, 1988). *Lactobacillus brevis* can produce 2‐acetyltetrahydropyridine (ACTPY), a compound that confers a *mousy* aroma in wines (Costello et al., 2001). *Geranium* aroma taint is a concern in wines that can result from the growth of lactic acid bacteria, specifically species within the genus *Pediococcus* (Sponholz, 1993). Besides these known wine fault compounds listed above, growth of these microorganisms is known to alter other components that can change wine taste (Du Toit & Pretorius, 2000), and a nonselective method can be useful to detect overall changes in a complex mixture like wine (Vlasov et al., 2005).

Early detection of microbial spoilage could allow winemakers to identify and minimize the progression of problematic metabolites. Over time, methodology to detect wine faults has evolved. The e‐tongue is a fairly new instrument that has been used to detect the development of red wine faults (Paup et al., 2021). This e‐tongue is equipped with an array of seven cross‐selective chemical sensors that can detect overall changes in all soluble organic and inorganic compounds in liquid matrices (Diako et al., 2017). This tool mimics human taste without the sensory fatigue caused by taint or trigeminal stimulation from beverages that contain alcohol and phenolics (Louw et al., 2014). Thus, the e‐tongue has the potential to complement sensory analysis of wine (Louw et al., 2014; Paup et al., 2021). The e‐tongue results were promising for detecting changes in wines spiked with soluble organic compounds including 4‐ethylcatechol, a compound that can cause *savory* aroma and *horsey* flavor in wine (Botha, 2010) at concentrations of 493 µg/L, below the subthreshold of human detection (Diako et al., 2017). In another wine study, the e‐tongue discriminated Merlot wine faults caused by spoilage microorganisms 7 days before sensory panelists detected differences (Paup et al., 2021).

Given that the e‐tongue has been seldom used for the detection of white wine faults, the objective of this study was to assess the capability of the e‐tongue in tracking chemical changes (potentially faults) from microbial spoilage in Riesling wines. Previous work completing sensory profiling of white wines found that rate‐all‐that‐apply (RATA) and descriptive analysis (DA) both had similar capabilities in sample discrimination (Danner et al., 2018). Due to the advantage of reduced training time needed with RATA than DA, this method is more cost‐effective for wine sensory analysis (Montero et al., 2020). Riesling wines were inoculated with white wine Washington‐state isolated cultures of *W. anomalus*, *A. aceti*, *L. brevis*, or *P. parvulus* and stored for 42 days at 22.3°C. Wines were assessed every 7 days with the e‐tongue and a RATA sensory panel.

## 2 MATERIALS AND METHODS

### 2.1 Materials

Modified Rogosa (MR) agar was made using tryptone, peptone, yeast extract, glucose, apple juice, Tween 80, and distilled water. Tryptone and yeast extract were obtained from Becton, Dickinson and Company. Peptone was obtained through VWR Chemicals. Agar and tween 80 were obtained through Acros Organics. Dextrose was obtained through Thermo Fisher Scientific. Wallerstein differential medium (WL) was obtained from Difco. Sodium chloride, hydrochloric acid, and sodium‐l‐glutamate solutions were obtained from Alpha Mos for e‐tongue conditioning, calibration, and diagnostics. Carlo Rossi Rhine White Wine was obtained. Chardonnay and Wine Faults kits were obtained for sensory panel training from Wine Awakenings.

### 2.2 Yeast starter cultures

*Wickerhamomyces anomalus* P01A017 culture isolated from Washington (WA) state wine was used (Aplin et al., 2019). The yeast culture, *W. anomalus*, was grown in WL broth as described by Paup et al. (2021). Yeast was grown on WL agar and incubated at 28.0°C for 7 days. A single colony of yeast was transferred to 50 mL of WL broth and incubated for 7 days at 28.0°C. Yeast (1 mL) was added to WL broth containing 5% ethanol (v/v) and was incubated at 28.0°C for 7 days. Culture (1 mL) was then transferred to WL broth containing 10% ethanol (v/v) and was incubated at 28.0°C for 7 days. Culture (1 mL) was then transferred to WL broth containing 12.5% ethanol (v/v) and was incubated at 28.0°C for 7 days. Cells were harvested by centrifuging at 1507 × *g* for 10 min and washed with 0.2 M Na_2_HPO_4_ buffer. Yeast cultures suspended in buffer were added to the wine at a concentration of 1 × 10^4^ CFU/mL.

### 2.3 Bacteria starter cultures

Bacteria cultures *A. aceti* (Takush & Osborne, 2011), *L. brevis*, and *P. parvulus* WS‐29A isolated from WA state wines were used (Paup et al., 2021). Bacterial cultures were grown in MR broth as described by Paup et al. (2021). Bacterial cultures were grown on MR agar and incubated at 28.0°C for 7 days. A single colony of *A. aceti*, *L. brevis*, and *P. parvulus* cultures was individually transferred to 50 mL of MR broth and incubated at 28.0°C for 7 days. The three bacterial cultures (1 mL) were each transferred to 50 mL of MR broth containing 5% ethanol (v/v) and incubated at 28.0°C for 7 days. These were then transferred to 50 mL of MR broth containing 10% ethanol (v/v) and incubated at 28.0°C for 7 days. The three bacterial cultures (1 mL) were transferred to 50 mL of MR broth containing 12.5% ethanol (v/v) and incubated at 28.0°C for 7 days. Cells were harvested by centrifuging at 1507 × *g* for 10 min and washed with 0.2 M Na_2_HPO_4_ buffer. Bacterial cultures suspended in buffer were added to the wine at a concentration of 1 × 10^4^ CFU/mL.

### 2.4 Control and treatments

Riesling wine produced in the Columbia Valley (*n* = 98; screw‐capped and in 750‐mL Alsace‐shaped green bottles) (Laposa et al., 2023) were obtained from the 2020 vintage (Paterson, WA, USA). Cultures of *W. anomalus* P01A017 (Aplin et al., 2019), *A. aceti* (Takush & Osborne, 2011), *L. brevis* (Paup et al., 2021), and *P. parvulus* WS‐29A (Paup et al., 2021) at 1 × 10^4^ CFU/mL were inoculated directly into Riesling wine bottles (*n* = 14 bottles per treatment, two replicates at each sampling point). Due to the oxygen requirement of *A. aceti* for growth (Arai et al., 2016), cultures of *A. aceti* were inoculated into additional bottles of Riesling that were stored halfway unscrewed. For control samples, uninoculated with yeast nor bacteria culture, sealed bottles (*n* = 14 bottles of sealed control) were stored alongside the inoculated bottles. An additional uninoculated control (*n* = 14) was left halfway unscrewed to mimic the second *A. aceti* treatment. Control and treated wines were stored at 22.3°C in a dark room for 42 days, and temperature was monitored using an FKM 7830D infrared thermometer (FKM).

### 2.5 Plate counts

At each storage timepoint (0, 7, 14, 21, 28, 35, and 42 days from initial inoculation), the culturability of the microorganisms in each treatment was monitored by plate counts. Yeast and bacterial cultures were monitored in replicate on WL and MR agars, respectively. Cultures were incubated at 30.0°C for 7 days prior to plate counting.

### 2.6 E‐tongue

Wines were analyzed using a potentiometric e‐tongue (Astree II electronic tongue unit; Alpha MOS) within 1 h of sensory evaluation at each sampling point. The potentiometric e‐tongue used in this study utilizes seven cross‐selective sensors that are coated in a membrane and are designed to measure the potential difference between ions and molecules in a sample compared to a reference electrode (Diako et al., 2017). The reference electrode contained a calibrated solution of Ag/AgCl as per manufacturer recommendations (Alpha MOS, 2011). Instrument preparation and sampling procedures were completed following manufacturer procedures including calibration, conditioning, and diagnostic procedures as previously described by Alpha MOS (2011). For each e‐tongue analysis, samples from two bottles of each treatment were analyzed, in addition to a reference white wine sample (Carlo Rossi Rhine) to account for instrument variability over time (Paup et al., 2021). Each set of wines was separated with a high‐purity water (type I) 10‐s sensor cleaning. Results were analyzed with AlphaSoft software (ver. 12; Alpha MOS).

### 2.7 Sensory evaluation assessment

The present study protocol received approval from the WSU (Washington State University) Institutional Review Board (IRB) for conducting with human subjects under IRB #19148‐001.

#### 2.7.1 Training

Sensory panelists (*n* = 13, eight women and five men), aged 25–78 (mean age = 42.9 years), participated in a RATA panel. For this sensory panel, panelists attended two 1‐h training sessions. Some of the compounds that are linked to spoilage in wine can contribute to wine complexity at low concentrations (Padilla et al., 2016), and to address this, both positive and negative spoilage attributes were used during the training of RATA sensory panelists. Panelists were trained on wine spoilage and non‐spoilage aroma attributes including *apple/pear*, *honey*, *baking spices*, *mousy*, *vegetal*, *peach*, *vinegar/nail polish remover*, *floral* (*geranium*), and *butter* (Table 1). Additionally, panelists were introduced to RATA methodology during training sessions and completed practice assessments of different wine samples using a RATA ballot on Compusense. This list of attributes was developed from Willwerth et al. (2015), a study that conducted DA on Riesling wines. Through training, panelist performance was assessed by presenting blind replicates of the same wine sample at each training session. Feedback was provided to the panelists in the form of panel means, and trained panelists were asked to examine their relationship between their attribute intensities to the panel mean. Panelists with intensity means ±2 standard deviations from the panel mean were asked to practice with the standard and re‐assess the sample. This specific feedback about their own performance was provided after both training sessions.

**TABLE 1:** Standard composition of aroma attribute references used in the training of RATA (rate‐all‐that‐apply) sensory panelists.

Aroma attribute	Standard composition
Apple/pear	5 g of Bartlett pear/5 g of Granny Smith apple/5 g of Red Delicious apple in 30 mL of base wine^a^, ^b^
Vegetal (low)	5 mL of canned green bean juice in 30 mL of base wine^b^
Vegetal (high)	42 mL of canned green bean juice in 30 mL of base wine
Honey	6 g of honey in 30 mL of base wine^b^
Baking spices	0.1 g of cinnamon and 0.1 g of nutmeg in 30 mL of base wine^b^
Butter	∼0.05 g of butter standard^c^ in 30 mL of base wine
Vinegar/nail polish remover	∼1 g of vinegar standard and ∼1 g of nail polish remover standard^d^ in 30 mL of base wine
Banana	∼0.05 g of 99.9% isoamyl acetate in 30 mL of base wine
Mousy	25 g of mouse bedding that had been in contact with mice
Floral (geranium)	One drop of geranium standard oil^d^ in 30 mL of base wine

^a^ Base wine—Carlo Rossi Rhine white wine.

^b^ Standard composition prepared as described by Willweth et al. (2015).

^c^ Wine Awakenings Chardonnay kit.

^d^ Wine Awakenings Wine Faults kit.

#### 2.7.2 Sensory evaluations

All sensory evaluation assessments were completed under white lighting in partitioned booths at the WSU Sensory Science Center. Semitrained panelists assessed faulted wine at each storage timepoint (0, 7, 14, 21, 28, 35, and 42 days from initial inoculation). Wines were poured (20 mL) 1 h prior to sensory evaluation in ISO (International Organization for Standardization) glass, labeled with three‐digit codes, and covered with petri dish. Wines were served to panelists at ambient temperature (∼22°C).

Panelists evaluated samples one at a time in a randomized order. Each microorganism treatment was analyzed in replicate, sampled from two separate wine bottles. Panelists were required to take a 30‐s break between samples, and a 2‐min break midway through the sample set. Leftover wine from each replicate treatment bottle during sensory analysis was saved for e‐tongue analysis by immediately flushing bottles with N_2_ gas and running e‐tongue analysis within 1 h of sensory analysis. Thus, with five treatments and two controls in replicate, a total of 14 samples were assessed every 7 days. Panelists rated the intensity of selected aroma terms using the RATA scale (1 = low, 2 = medium, and 3 = high). A 30‐s break was provided between each sample, and a 2‐min break was presented after the panelists evaluated seven samples to limit sensory fatigue. 

### 2.8 High‐performance liquid chromatography

Wines were analyzed after 0, 14, 21, and 42 days of storage at 22.3°C for glucose, malic acid, fructose, succinic acid, glycerol, acetic acid, and ethanol concentrations using high‐performance liquid chromatography (HPLC)–diode array detection (DAD) (Charoenchai et al., 1998) and external standard method. Glucose and fructose were analyzed to assess microbial growth in the wines. For peak quantification, standard curves were created for each chemical component, and two replicates were analyzed for each concentration. As fructose and malic acid coeluted, a third standard curve combining both compounds, at concentrations from their respective standard curves, was created to differentiate these compounds (Eyéghé‐Bickong et al., 2012). Chemicals were all obtained from Fisher Scientific.

### 2.9 Data and statistical analyses

XLSTAT 2017 (Addinsoft) was used for sensory data analysis. Sensory data were analyzed by treating the RATA scale as continuous data and expanding the scale to 4 points (0 = *absent*, 1 = *low*, 2 = *medium*, and 3 = *high*) (Montero et al., 2020). Two‐way analysis of variance (ANOVA) was used to assess consistency in aroma intensity ratings among replicates at each session.  A three‐way ANOVA was conducted to determine the effect of storage time, wine replicate, and panelist on aroma attribute intensity. Mean separations were completed using Tukey's honest significant difference (HSD) test with significance defined at *p* ≤ 0.05. For each spoilage organism, principal component analysis (PCA) was used to visualize differences in samples over time. Statistical comparisons were not made between the wine microorganism treatments since each microorganism‐inoculated wine was expected to produce different wine faults.

E‐tongue data were analyzed using Astree Alphasoft to calculate the discrimination index (DI) and conduct PCA. DI is a measure of overlap among samples and distance between samples. A negative DI indicates overlap, and a positive DI indicates separation. To determine when the e‐tongue could begin detecting significant differences in sealed inoculated wines, e‐tongue results were defined as significant when the DI was high (DI ≥ 80%) when comparing sealed inoculated wine sample at all timepoints to wine that was inoculated with cultures and immediately analyzed using the e‐tongue after inoculation at time zero (Alpha MOS, 2011). To determine when the e‐tongue could begin detecting significant differences in unsealed inoculated wines, the e‐tongue results were defined as significant when the DI was high when comparing unsealed inoculated wine samples at all timepoints to unsealed uninoculated control wine sampled at day 42 of storage. This was to determine that changes happening in the unsealed inoculated wines were due to more than just oxidation. Additionally, both the sealed and unsealed controls from all sampling timepoints (*t* = 0, 7, 14, 21, 28, 35, and 42) were plotted using PCA to determine that significant changes did not occur in each control. Each wine treatment was analyzed from two replicates and sampled from two separate wine bottles.

Sensory and e‐tongue results were compared to determine which methods could detect differences in the wine treatments after the various storage period. Results were analyzed using Pearson's correlation analysis to determine significant relationships between the two methods. Correlation analysis was completed using XLSTAT 2017. Significance was defined at *p ≤ *0.05.

## 3 RESULTS AND DISCUSSION

### 3.1 Microbial analysis

Growth of these four microorganisms in all the treatments could not be determined by plate count method. However, glucose and fructose levels were significantly reduced in wines inoculated with *A. aceti*, *L. brevis*, and *P. parvulus* (Table S1), suggesting that microbial metabolic activity had occurred despite the lack of growth on plates. Significant sugar reduction did not occur in wines inoculated with *W. anomalus*, although acetic acid concentration in these wines increased over time. Based on these chemical changes in the wine, presumption was made that all the microorganisms were metabolically active, although further microbial enumeration methods would have been necessary to confirm this.

A few reasons might explain this seemingly low microbial growth. Plate count method as well as the media used may have been inadequate of assessing growth in this study. Plate counting is less sensitive than other enumeration methods including direct epifluorescence technique (DEFT) (Millet & Lonvaud‐Funel, 2000). Acetic acid bacteria and lactic acid bacteria plate count enumeration can differ by a factor of 10^2^ or more compared to DEFT (Millet & Lonvaud‐Funel, 2000). Additionally, previous literature has found that wine microorganisms can survive in a viable but not culturable (VBNC) state (Millet & Lonvaud‐Funel, 2000). The environmental conditions of the wine, including ethanol and sulfite, may have been stressors that induced the VBNC state. Millet and Lonvaud‐Funel (2000) found that after sulfiting red wine, the population of viable lactic acid bacteria able to grow on nutrient agar plates was reduced drastically, but the population remained viable at metabolizing the fluorescent dye when performing the DEFT method. Ethanol and sulfite both have an inhibitory effect on cell metabolic activity (Carreté et al., 2002). An approach to overcome this effect could have included a pre‐enrichment step, which would have allowed the microorganisms to recover from the stress of the ethanol and the sulfite before being plated for enumeration (Bae et al., 2006).

### 3.2 E‐tongue results

**FIGURE 1:**
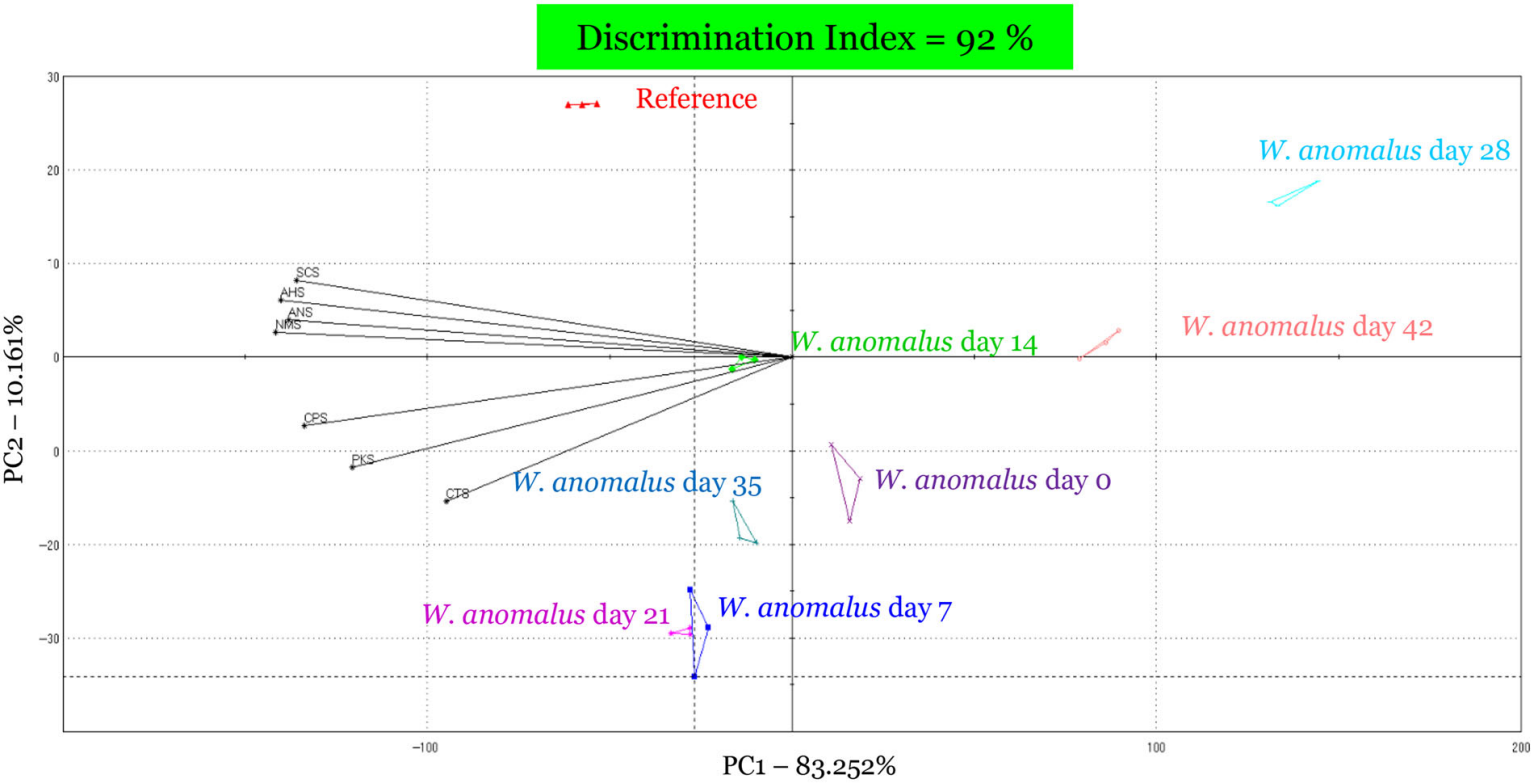
Principal component (PC) analysis responses to a Riesling wine inoculated with *Wickerhamomyces anomalus* as assessed by the e‐tongue. Inoculated wine samples were stored at 22.3°C for 42 days and sampled weekly. The colored clusters on the plot represent the time the inoculated sample was stored prior to analysis. Carlo Rossi Rhine white wine was used as an instrument reference to account for instrument variation. E‐tongue sensor vectors are in black. The sensors are indicated by SCS, AHS, NMS, PKS, ANS, CPS, and CTS.

**FIGURE 2:**
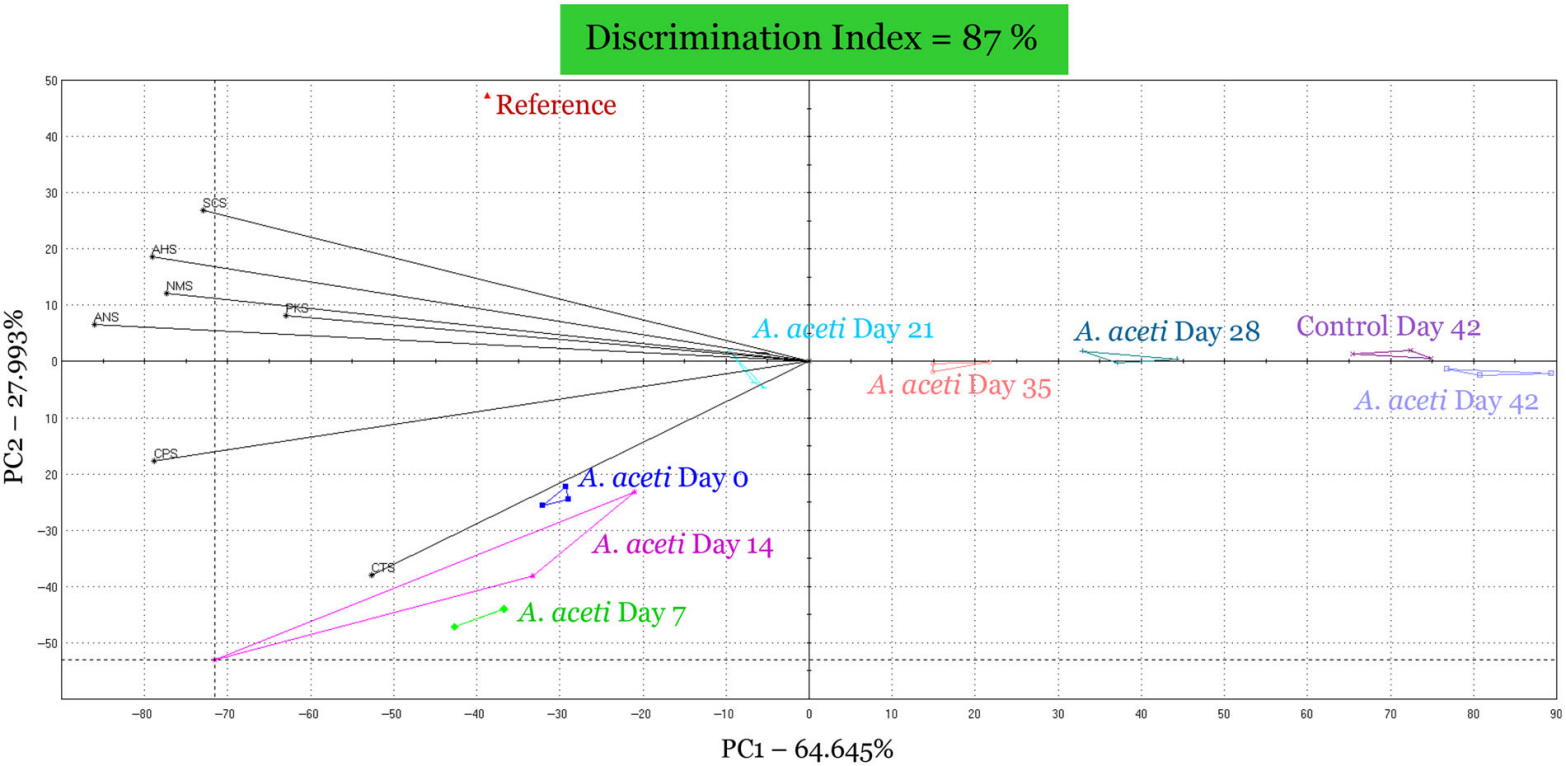
Principal component (PC) analysis responses to a Riesling wine inoculated with *Acetobacter aceti* stored partially unscrewed as assessed by the e‐tongue. Inoculated wine samples were stored at 22.3°C for 42 days and sampled weekly. The colored clusters on the plot represent the time the inoculated sample was stored prior to analysis. Carlo Rossi Rhine white wine was used as an instrument reference to account for instrument variation. E‐tongue sensors (in black) are indicated by SCS, AHS, NMS, PKS, ANS, CPS, and CTS. Partially unscrewed control wine at day 42 was included to show that changes in the partially unscrewed inoculated wines were not exclusively due to oxidation.

**FIGURE 3:**
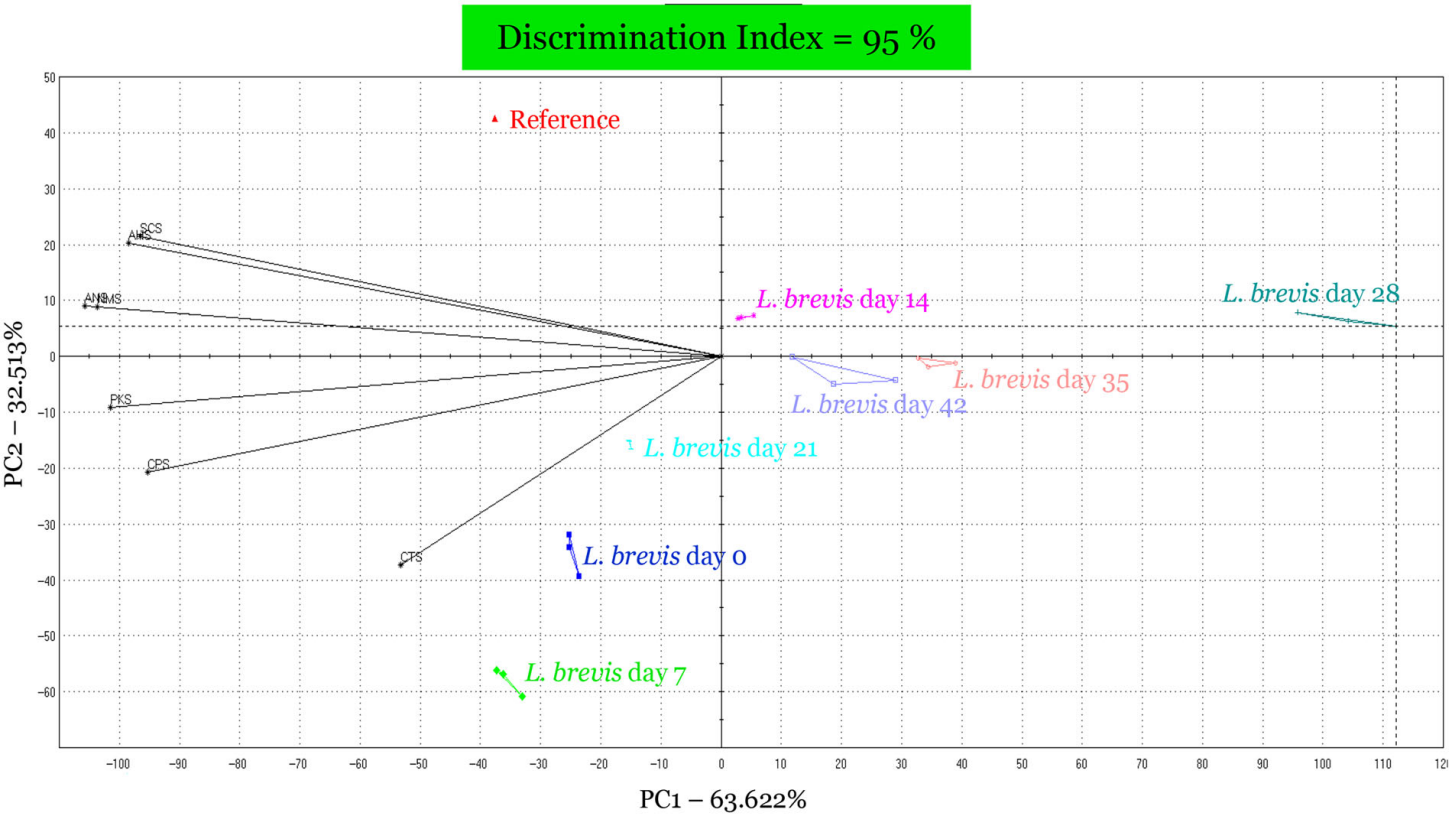
Principal component (PC) analysis responses to a Riesling wine inoculated with *Lactobacillus brevis* as assessed by the e‐tongue. Inoculated wine samples were stored at 22.3°C for 42 days and sampled weekly. The colored clusters on the plot represent the time the inoculated sample was stored prior to analysis. Carlo Rossi Rhine white wine was used as an instrument reference to account for instrument variation. E‐tongue sensor vectors are in black. The sensors are indicated by SCS, AHS, NMS, PKS, ANS, CPS, and CTS.

**FIGURE 4:**
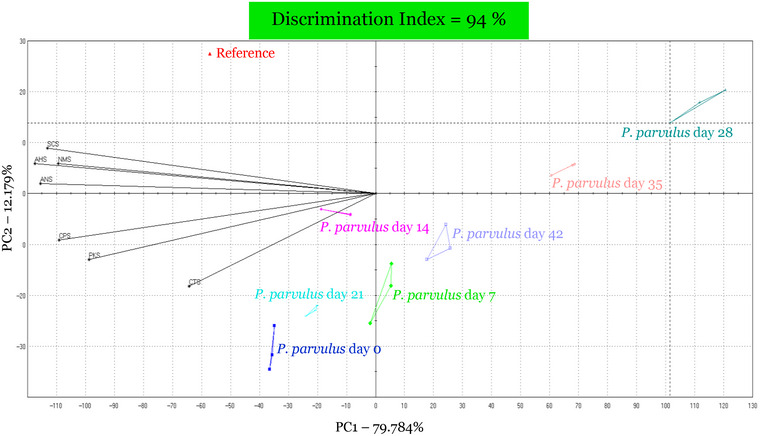
Principal component (PC) analysis responses to a Riesling wine inoculated with *Pediococcus parvulus* as assessed by the e‐tongue. Inoculated wine samples were stored at 22.3°C for 42 days and sampled weekly. The colored clusters on the plot represent the time the inoculated sample was stored prior to analysis. Carlo Rossi Rhine white wine was used as an instrument reference to account for instrument variation. E‐tongue sensor vectors are in black. The sensors are indicated by SCS, AHS, NMS, PKS, ANS, CPS, and CTS.

The e‐tongue discriminated among all inoculated wines and control wine with high certainty (DI ≥ 80%) after 7 days of storage (*W. anomalus* in Figure 1, *A. aceti* in Figure 2, *L. brevis* in Figure 3, and *P. parvulus* in Figure 4). After 7 days of storage, wines inoculated with *A. aceti* had a DI of 98% when control (uninoculated) wine stored partially unscrewed was included in the PCA model. The DI dropped slightly as the storage study progressed, and as additional samples were included in the PCA model. When inoculated wines sampled at all timepoints and a partially unscrewed control from the final sampling point (day 42) were included in the PCA model, the e‐tongue had a DI of 87% (Figure 2).

In the PCA plot, the partially unscrewed control wine stored for 42 days was close in proximity to wine inoculated with *A. aceti* stored partially unscrewed for 42 days (Figure 2). Notably, the partially unscrewed control wine that was sampled at the same timepoint as the *A*. *aceti‐*inoculated wine fell in close proximity to inoculated wine at each respective sampling time. For example, wine inoculated with *A. aceti* stored for 35 days was close in proximity to the partially unscrewed control wine stored for 35 days. This proximity suggests that these samples had a similar chemical composition of soluble compounds. To illustrate separation among the inoculated samples in Figure 2, only the partially unscrewed control wine from the final analysis point (day 42) was included to show that the changes occurring in these partially unscrewed inoculated wines were due to the development of wine faults and not due to air exposure. Across the 42‐day storage time, the e‐tongue poorly discriminated the chemical components of wine inoculated with *A. aceti* and stored sealed; the result was a negative DI.

An increase in DI occurred in wines inoculated with *W. anomalus* or *L. brevis* over time. For wines inoculated with *W. anomalus*, the DI was 86% when compared to control wine after 7 days of storage, increasing to 92% when including inoculated wine from all sampling points in the PCA model (Figure 1). Similarly, wines inoculated with *L. brevis* had a DI of 94% after 7 days of storage when compared to the control wine, and the DI increased to 95% when including inoculated wine from all sampling points in the PCA model (Figure 3).

By contrast, the DI of wines inoculated with *P. parvulus* slightly decreased over time. After 7 days of storage, the DI of wines inoculated with *P. parvulus* was 96% when control (sealed) wine was included in the PCA model. The DI decreased to 94% when inoculated wines from all sampling points were included in the PCA model (Figure 4).

The results of the control wines at the different timepoints were also examined using the e‐tongue. When sealed control wines from all sampling points (*t* = 0, 7, 14, 21, 28, 35, and 42 days) were included in the PCA model, the e‐tongue could not discern among these samples; the result was a negative DI (−13%). Similarly, the uninoculated control wine stored partially unscrewed also had a negative DI (−31%), indicating that there was overlap on the PCA plot among both controls sampled at different timepoints. Specifically, there was overlap between the partially unscrewed control wine on sampling days 35 and 42, indicating that these wines were similar in soluble compounds, though specific compound differences cannot be determined with the e‐tongue. When partially unscrewed control wine sampled on day 42 was removed from the PCA plot (the PCA model contains partially unscrewed control wine from *t* = 0, 7, 14, 21, 28, and 35 days), the DI was high (98%). These results would suggest that the e‐tongue also holds promise for detecting oxidation in white wines.

To further understand the changes in the e‐tongue sensor signal intensities with storage time, a one‐way ANOVA was conducted to determine the effect of storage time on sensor signal intensity, and mean separations were completed using Tukey's HSD test. For each wine microorganism, the intensity of each individual sensor was affected by storage time (Table S2).

These results can be interpreted by considering the e‐tongue sensors and how they measure a sample. The e‐tongue sensors can detect overall differences in soluble organic and inorganic compound makeup of a sample but are nonselective for measuring specific analytes in a solution (Vlasov et al., 2005). The sensors have low selectivity and high cross‐sensitivity with high stability, making them an appropriate method for pattern recognition and multivariate calibration during data analysis (Vlasov et al., 2005). Thus, a high DI would indicate that there are overall differences in soluble chemical makeup between samples, though specific differences in composition cannot be determined with the e‐tongue.

Oxidation most likely contributed to changes occurring in wines inoculated with *A. aceti* stored partially unscrewed. For this discussion, only soluble compound change will be considered. Enzymatic oxidation of monophenol and monocatechol produces brown polymers that contribute to soluble chemical changes within oxidized wines (Li et al., 2008). Additionally, the wines used in the present study contained free SO_2_ (12.4 mg/L), which can help prevent unpleasant sensory changes from oxidation in wines by reacting with carbonyl compounds to produce bisulfite adducts (Oliveira et al., 2011). Thus, changes detected by the e‐tongue in wines stored partially unscrewed (both inoculated and control) were most likely a result of chemical changes occurring in the wine from oxygen exposure.

However, chemical changes occurring in the wines inoculated with *A. aceti* stored partially unscrewed were not exclusively due to oxidation. A partially unscrewed control was included in the PCA model to determine if differences detected by the e‐tongue in wines stored partially unscrewed and inoculated with *A. aceti* could be attributed to changes other than oxidation. Since good separation did occur when including both partially unscrewed inoculated and partially unscrewed uninoculated wine (DI = 87%) (Figure 2), this would imply that changes within these wines were due to more than just oxidation itself. Additional chemical changes happening in these wines were most likely caused by microbial activity. Significant reduction in glucose and fructose occurred in wines inoculated with *A. aceti* over time (Table S1), which also contributed to the differences in the samples and control detected by the e‐tongue with time.

Differences were also detected by the e‐tongue for sealed wine treatments including wines inoculated with *P. parvulus*, *L. brevis*, or *W. anomalus*. Changes detected by the e‐tongue in wines inoculated with *P. parvulus* or *L. brevis* could have been attributed to significant reduction in glucose and fructose (Table S1). A significant increase in acetic acid concentration could have allowed the e‐tongue to detect differences in wines inoculated with *W. anomalus*. Additional chemical changes within the sealed wine treatments could have resulted from aging (Table S1).

The findings from this study can be supported by the results of previous studies that used the e‐tongue for the early detection of wine faults. A study with similar methodology to the present study found that the e‐tongue detected changes in spoilage‐organism‐inoculated Merlot wines after 21 days of storage prior to a sensory panel at day 28 of storage (Paup et al., 2021). However, the sensory panel used for the detection of Merlot wine faults was not a semitrained panel; therefore, the present study provides additional support for the e‐tongue being a sensitive method. In addition, previous work suggests that the e‐tongue may also be useful for detecting changes that occur after the addition of certain soluble organic compounds (Diako et al., 2017). This study found that the e‐tongue could discern among control Merlot wine and Merlot wines containing subthreshold concentrations of 4‐ethylcatechol, a soluble organic compound (Diako et al., 2017). Further research is needed to fully assess the e‐tongue's capabilities in detecting wine compounds.

### 3.3 Sensory results

Significant changes in spoilage aroma attributes were detected by the semitrained sensory panel across the 42‐day storage time. In particular, the aroma intensity of *vinegar/nail polish remover* increased significantly in wines inoculated with *W. anomalus* and *L. brevis* with respect to time. Intensity of *vinegar/nail polish remover* in wines inoculated with *W. anomalus* significantly increased on day 42 (0.92; 1 = low to 3 = high) when compared to control wine sampled on day 0 (0.46) (Table 2). Similarly, the intensity of *vinegar/nail polish remover* in wines inoculated with *L. brevis* increased on day 42 (1.00) when compared to control wine sampled on day 0 (0.58) (Table 2). Wines inoculated with *A. aceti* stored partially unscrewed had an overall increase in *vinegar/nail polish remover* with time, with the intensity of the aroma reaching 1.19 on day 42 of storage, but this increase was not significant. Overall, the intensity of aroma attribute *vinegar/nail polish remover* increased to approximately a “low” intensity on the RATA scale for wines inoculated with *W. anomalus*, *L. brevis*, and *P. parvulus* after 42 days of storage.

**TABLE 2:** Aroma intensity responses to a Riesling wine inoculated with *Wickerhamomyces anomalus*, *Acetobacter aceti*, *Lactobacillus brevis*, or *Pediococcus parvulus* stored for 42 days at 22.3°C as assessed by a rate‐all‐that‐apply (RATA) sensory panel using a 3‐point scale.

		Storage time (days)							
Microorganism	Aroma attribute	0	7	14	21	28	35	42	*p*‐value (storage time)
*W. anomalus*	Vinegar/nail polish remover	0.46**bc**	0.70**ab**	0.46**bc**	0.73**ab**	0.23**c**	0.71**ab**	0.92**a**	0.052
*A. aceti*	Vinegar/nail polish remover	0.77**abc**	0.95**ab**	0.38**c**	0.68**bc**	0.73**bc**	0.58**bc**	1.19**a**	**0.022**
*L. brevis*	Mousy	0.00**b**	0.05**ab**	0.00**b**	0.00**b**	0.00**b**	0.00**b**	0.12**a**	**0.042**
	Vinegar/nail polish remover	0.58**bc**	0.50**c**	0.42**c**	0.96**ab**	0.36**c**	0.42**c**	1.00**a**	**0.004**
*P. parvulus*	Butter	0.00**b**	0.00**ab**	0.08**ab**	0.091**ab**	0.05**ab**	0.21**a**	0.19**a**	0.208
	Banana	0.12**b**	0.30**ab**	0.17**ab**	0.273**ab**	0.14**ab**	0.04**b**	0.39**a**	0.143
Control (sealed)	Vinegar/nail polish remover	0.73a	0.89a	0.72a	0.85a	0.55a	0.46a	0.65a	0.495
	Banana	0.35a	0.42a	0.22a	0.07a	0.23a	0.19a	0.19a	0.584
Control (unsealed)	Vinegar/nail polish remover	0.92**a**	0.25**c**	0.80**ab**	0.57**abc**	0.42**bc**	0.69**ab**	0.50**bc**	**0.025**

*Note*: The *p*‐value listed in a column represents the significance of storage time on the concentration of that compounds as determined using analysis of variance. A bolded different letter in a row represents a significant difference as determined using Tukey's HSD (*p* ≤ 0.05).

Additional changes in aroma were noted among the inoculated wines. Wines inoculated with *L. brevis* had a significant increase in *mousy* aroma after 42 days of storage. Additionally, wines inoculated with *P. parvulus* had significant increases in *butter* and *banana* aromas. Significant increases in *butter* aroma began after 35 days of storage, and on day 42, *butter* aroma remained significantly greater than the intensity observed at day 0. Significant increase in *banana* aroma intensity began after 42 days of storage.

Sealed uninoculated control wines did not have any significant changes in aroma attribute during storage when comparing wine sampled at day 0 to day 42. Unsealed uninoculated control wines had a significant decrease in *vinegar/nail polish remover* aroma when comparing unsealed wine sampled at storage days 0 and 42.

Increase in *vinegar/nail polish remover* intensity in wines inoculated with *W. anomalus* is supported by previous research. Muscat wines fermented with *W. anomalus* have previously been characterized as having higher levels of fruity acetate esters including isoamyl acetate, ethyl acetate, and 2‐phenylethylacetate (Viana et al., 2008). This may explain why there was a significant increase in *vinegar/nail polish remover* aroma beginning on day 42 of storage. In the present study, *banana* aroma did not increase significantly during storage, and this may be due to the ethanol intolerance of *W. anomalus* (Walker, 2011). While in the present study, the *W. anomalus* cultures were grown up in media with increasing concentrations of ethanol, the environment of the research Riesling wine may still have been not ideal for the cells to produce sufficient acetate esters to impact *banana* aroma. Changes in the aroma profile of wines inoculated with *W. anomalus* can be attributed to significant increase in acetic acid, and potentially an increase in ethyl acetate in the wines.

Significant increase in *mousy* and *vinegar/nail polish remover* aroma intensity in wines inoculated with *L. brevis* is supported by what has been observed in previous research. Previous work found, when examining lactic acid bacteria growth in ethanolic Doradillo grape juice medium, that *L. hilgardii* and *L. brevis* produced the highest concentration of ACTPY and also produced moderate concentrations of 2‐acetyl‐1‐pyrroline (ACPY) and 2‐ethyltetrahydropyridine (ETPY) (Costello et al., 2001). ACTPY, ACPY, and ETPY are all *N*‐heterocycles and are associated with mousy off‐odor in wines (Herderich et al., 1995; Strauss & Heresztyn, 1984; Tucknott, 1977). A previous study found that Chardonnay wine fermented with *L. brevis* produced a high concentration of acetic acid (Gao et al., 2002). Overall, the sensory results for wines inoculated with *L. brevis* are supported by what has been found by others.

Wines inoculated with *P. parvulus* had a significant increase in *butter* aroma intensity during storage, which can also be supported by previous literature. *Pediococcus* spp. have previously been reported to produce excess amounts of diacetyl, a compound with a butter aroma (Wade et al., 2019). Previous work by Strickland et al. (2016) found that certain strains of *P. parvulus* can produce high concentrations of diacetyl in Pinot noir wines. The sensory threshold of diacetyl varies based on the wine type, but Martineau et al. (1995) found that Chardonnay wines had a lower sensory threshold (0.2 mg/L) when compared to Pinot noir (0.9 mg/L) and Cabernet Sauvignon (2.8 mg/L) wines. Generally, the sensory threshold for diacetyl is lower in white wines than in red wines (Martineau et al., 1995).

### 3.4 Comparison of e‐tongue and sensory results

Significant correlations (Table 3) were noted among some of the wine samples when comparing e‐tongue DIs and aroma intensity results. In wines inoculated with *W. anomalus*, a significant relationship existed between the pattern DIs produced by the e‐tongue and *vegetal* aroma intensity (−0.974), indicating that as DI increased, *vegetal* aroma decreased. Vegetal is an aroma that has previously been used to describe microbially uncontaminated Riesling wines by a DA panel (Schüttler et al., 2015). A reduction in *vegetal* aroma as observed in the present study may indicate that the Riesling wines might be an indication of the wine losing its characteristic aroma, and the e‐tongue can detect these changes by producing higher pattern DIs (Paup et al., 2021).

**TABLE 3:** Significant relationships determined with Pearson correlation analysis between variables including aroma attribute intensities assessed by a rate‐all‐that‐apply (RATA) panel, e‐tongue pattern discrimination indices (DIs), and time (storage weeks) for inoculated Riesling wine samples.

Wine treatment	*W. anomalus*	*P. parvulus*
Variables	*Vegetal* aroma	*Butter* aroma
Pattern DI	**−0.974**	
Fructose (g/L)		**−0.991**
Ethanol (g/L)		**−0.979**

*Note*: Values in bold denote a significant relationship between two variables (*p* ≤ 0.05). Other comparisons were made, but only these three variables and two attributes from two organisms were significantly correlated.

Significant relationships also existed in wines inoculated with *P. parvulus* (Table 3). A significant relationship was present between fructose concentration (Table S1) and *butter* aroma intensity (−0.991), indicating that as fructose concentrations decreased, *butter* aroma increased. Most strains of *P. parvulus* can metabolize and use fructose as a carbon source (Velasco et al., 2007), indicating that inoculated *P. parvulus* could have used fructose as a carbon source to produce diacetyl, although additional enumeration methods such as DEFT would be needed to confirm the growth of *P. parvulus*. Ethanol concentration and *butter* aroma intensity also had a significant negative relationship (−0.979), indicating that as ethanol concentration decreased, *butter* aroma intensity increased.

In this study, the e‐tongue was more sensitive than a semitrained RATA panel in detecting early changes in spoiled Riesling wines. The e‐tongue detected differences beginning on day 7 of storage for all wine treatments. In contrast, the semitrained RATA panel started to detect differences among the wine samples at day 35 of storage. These results suggest that the e‐tongue may be a more sensitive method for the early detection of wine faults in Riesling wines, detecting differences prior to a semitrained RATA sensory panel.

There are both advantages and disadvantages of using the e‐tongue as a method for wine quality monitoring. The low‐selectivity e‐tongue sensors provide a holistic measurement of a sample making them a good method for measuring overall differences. This is useful if the research question focuses on looking at differences among samples with respect to time for storage studies, and if one is completing sensory difference testing alongside e‐tongue analysis. However, the low‐selectivity sensors cannot measure specific analytes and their respective concentrations. Thus, the e‐tongue may not be the best method if the research question is centered around specific chemical changes occurring in a sample with time. Depending on the research question and the sample type, the e‐tongue has the potential to be a useful tool for determining overall differences among samples.

### 3.5 Limitations

This work contributed additional insight into the use of the e‐tongue for the early detection of microbial wine faults in white wine; previous work included the use of the e‐tongue for microbial wine fault detection in red wines (Paup et al., 2021). However, there were a few limitations with microbial growth in the present study. The microorganisms could have been incubated for longer than 7 days, as for lactic acid bacteria, they may need more than 10 days if the cells are stressed to form colonies (Millet & Lonvaud‐Funel, 2000). DEFT is a more sensitive method than plate counting, so this may have been a better way to track the microbial growth (Millet & Lonvaud‐Funel, 2000). Additionally, the cells could have been initially inoculated into an enriched broth to bring them out of a shocked state, prior to culturing these microbes on media for colony counts (Bae et al., 2006).

This study used the e‐tongue and RATA to describe changes in wine over time due to the presence of different microorganisms. Panelists evaluated only aroma and not the taste of the wines. If the assessors had also evaluated the wines for taste and flavor changes, this would have provided more information on metabolic activity of the microorganisms, including temporal monitoring changes such as a decrease in sweetness and increase in sourness. It would also have been useful to run a consumer panel to better relate these results to consumer perception, willingness to purchase, check‐all‐that‐apply, and overall liking.

Chemical measurements could also have been performed. To better track some of the components including soluble volatile compound changes in the wines with respect to time, GC–MS (gas chromatography–mass spectrometry) could have been used to quantify volatile metabolites during microbial growth. Monitoring specific volatile compounds such as ethyl acetate (Barbe et al., 2001), 2‐ethoxyhexa‐3,5‐diene, ETPY, ACTPY, and ACPY may have allowed tracking of *L. brevis* metabolic activity and correlating these concentrations to *mousy* aroma intensity (Costello et al., 2001). Isoamyl acetate and ethyl acetate, compounds with *banana* and *nail polish remover* aromas, respectively, could have been quantified to better understand metabolic activity in wines inoculated with *W. anomalus* (Padilla et al., 2018). These data would provide further insight into aroma intensity results. Correlation analysis between aroma intensity responses and GC–MS data could determine if significant relationships existed.

Another limitation of this study was the storage time (42 days). With a longer storage time, spoilage aroma attributes may have increased in intensities. The length of this storage study could have been extended to a year to further examine microbial growth. In addition, more frequent sampling could have been completed. Given the inconsistent increases and decreases in aroma intensity, this could have provided further insight into the RATA sensory results.

There are also limitations that must be considered when selecting the e‐tongue as a method for wine fault detection. The e‐tongue cannot detect specific chemical changes during the development of wine faults (Vlasov et al., 2005). If the winemaker suspects the development of a specific wine fault, other chemical analysis methods would be more appropriate. Additionally, since the e‐tongue cannot detect specific chemical changes in the wines, it is possible that dead biomass of the microorganisms had some effect on the changes in these wines that were detected by the e‐tongue.

One final limitation of this study was that the wines were not tasted by the trained RATA panel, and the e‐tongue primarily measures soluble organic compounds, which are primarily perceived in‐mouth. Sensory changes in the basic tastes were not tracked by the sensory panel. The microorganisms used in this study were not prepared in a food‐grade laboratory, and so the inoculated wines were limited to only being assessed for aroma changes.

## 4 CONCLUSION

Compared to RATA sensory analysis, the e‐tongue was more sensitive in detecting changes due to microbial spoilage in Riesling wine. Significant intensity increases in spoilage aroma attributes were not detected until day 35 for wines inoculated with *W. anomalus* and *L. brevis*. The e‐tongue discriminated among all wine treatments compared to respective control wine (sealed or partially unscrewed) beginning on day 7. DIs remained high for the remainder of the storage study in *W. anomalus*, *A. aceti* (stored unsealed), *L. brevis*, and *P. parvulus* treatments. Given the results of the present study, if winemakers suspect the development of a wine fault, the winemaker could send sample to a laboratory that provides e‐tongue analysis. Alongside sensory testing, the e‐tongue has the potential to be a useful tool for the early detection of microbial faults in white wines.

## AUTHOR CONTRIBUTIONS

**Rachel I. Potter**: Investigation; writing—original draft; methodology; validation; visualization; writing—review and editing; formal analysis; data curation. **Claire A. Warren**: Conceptualization; investigation; writing—original draft; methodology; writing—review and editing. **Jungmin Lee**: Conceptualization; investigation; writing—original draft; methodology; writing—review and editing; formal analysis. **Carolyn F. Ross**: Conceptualization; investigation; funding acquisition; writing—original draft; methodology; writing—review and editing; visualization; project administration; supervision; resources; data curation.

## CONFLICT OF INTEREST STATEMENT

The authors declare no conflicts of interest.

## ETHICS STATEMENT

This wine sensory panel was approved by the Institutional Review Board of WSU (IRB #19148‐001), with written informed consent obtained from all study participants.

## Supporting information

Supplemental Table 1: Chemical measurements on Riesling wines inoculated with Wickerhamomyces anomalus, Acetobacter aceti, Lactobacillus brevis, or Pediococcus parvulus stored on days 0 (control), 14, 21, and 42 of storage (22.3°C) as assessed using HPLC. The p‐value listed in a column represents the significance of storage time on the concentration of that compounds as determined using analysis of variance. A bolded different letter in a row represents a significant difference as determined using Tukey's HSD (p ≤ 0.05). 95% confidence intervals were calculated for each measurement.
Supplemental Table 2: Sensor signal measurements from the e‐tongue on Riesling wines inoculated with Acetobacter aceti, Pediococcus parvulus, Lactobacillus brevis, or Wickerhamomyces anomalus stored on days 0 (control), 7, 14, 21, 28, 35, and 42 of storage (22.3°C) as assessed using the e‐tongue. The p‐value listed in a column represents the significance of storage time on the intensity of that sensor signal as determined using analysis of variance. A bolded different letter in a column represents a significant difference in sensor signal intensity as determined using Tukey's HSD (p ≤ 0.05).

## Data Availability

The data that support the findings of this study are available from the corresponding author upon reasonable request.
