# Supplementary material for: Comparative assessment of Riesling wine fault development by the electronic tongue and a sensory panel
Source: J Food Sci. 2024 Mar 27;89(5):3006–18. doi: 10.1111/1750-3841.17036 (PMC13281126; doi:10.1111/1750-3841.17036)
Supplement: Supplementary file 1 — Supplemental Table 1: Chemical measurements on Riesling wines inoculated with Wickerhamomyces anomalus, Acetobacter aceti, Lactobacillus brevis, or Pediococcus parvulus stored on days 0 (control), 14, 21, and 42 of storage (22.3°C) as assessed using HPLC. The p‐value listed in a column represents the significance of storage time on the concentration of that compounds as determined using analysis of variance. A bolded different letter in a row represents a significant difference as determined using Tukey's HSD (p ≤ 0.05). 95% confidence intervals were calculated for each measurement. Supplemental Table 2: Sensor signal measurements from the e‐tongue on Riesling wines inoculated with Acetobacter aceti, Pediococcus parvulus, Lactobacillus brevis, or Wickerhamomyces anomalus stored on days 0 (control), 7, 14, 21, 28, 35, and 42 of storage (22.3°C) as assessed using the e‐tongue. The p‐value listed in a column represents the significance of storage time on the intensity of that sensor signal as determined using analysis of variance. A bolded different letter in a column represents a significant difference in sensor signal intensity as determined using Tukey's HSD (p ≤ 0.05). [file JFDS-89-3006-s001.docx]

**Supplemental Table 1:** Chemical measurements on Riesling wines inoculated with *Wickerhamomyces anomalus*, *Acetobacter aceti, Lactobacillus brevis*, or *Pediococcus parvulus* stored on days 0 (control), 14, 21, and 42 of storage (22.3°C) as assessed using HPLC. The *p*-value listed in a column represents the significance of storage time on the concentration of that compounds as determined using analysis of variance. A bolded different letter in a row represents a significant difference as determined using Tukey’s HSD (*p*≤0.05). 95% confidence intervals were calculated for each measurement.

|  |  | Storage Time (days) | | | |  |
| --- | --- | --- | --- | --- | --- | --- |
| Microorganism | Analyte | 0 | 14 | 21 | 42 | *p*-value (storage time) |
| *A. aceti* | **Glucose (g/L)** | **2.32 a** | **2.26 a** | **2.17 ab** | **1.93 b** | **0.021** |
|  | **Fructose (g/L)** | **20.21 a** | **20.20 a** | **19.15 b** | **15.90 c** | **<0.001** |
| *P. parvulus* | **Glucose (g/L)** | **2.32 a** | **2.15 b** | **2.18 b** | **2.21 b** | **0.016** |
|  | **Fructose (g/L)** | **20.21 a** | **18.98 b** | **18.90 b** | **18.03 b** | **0.011** |
| *L. brevis* | **Fructose (g/L)** | **20.21 a** | **19.20 d** | **20.04 b** | **19.58 c** | **<0.001** |
| *W. anomalus* | **Acetic acid (g/L)** | **0.10 c** | **0.10 c** | **0.12 a** | **0.11 b** | **0.002** |

**Supplemental Table 2:** Sensor signal measurements from the e-tongue on Riesling wines inoculated with *Acetobacter aceti,* *Pediococcus parvulus*, *Lactobacillus brevis*, or *Wickerhamomyces anomalus* stored on days 0 (control), 7, 14, 21, 28, 35, and 42 of storage (22.3°C) as assessed using the e-tongue. The *p*-value listed in a column represents the significance of storage time on the intensity of that sensor signal as determined using analysis of variance. A bolded different letter in a column represents a significant difference in sensor signal intensity as determined using Tukey’s HSD (*p*≤0.05).

| Microorganism | *Acetobacter aceti* | | | | | | |
| --- | --- | --- | --- | --- | --- | --- | --- |
|  | Sensor Identity | | | | | | |
| Storage Days | AHS intensity | ANS intensity | CPS intensity | CTS intensity | NMS intensity | PKS intensity | SCS intensity |
| 0 | 863.91 **e** | 1302.53**e** | 1083.69 **d** | 941.26 **e** | 1017.07 **d** | 1271.46 **c** | 1190.11 **d** |
| 7 | 677.29 **g** | 1114.36 **f** | 957.04 **e** | 1047.75 **d** | 748.29 **g** | 514.46 **g** | 983.89 **g** |
| 14 | 899.58 **d** | 1345.70 **d** | 1078.74 **d** | 773.39 **g** | 945.64 **f** | 564.75 **f** | 1179.74 **e** |
| 21 | 1710.24 **b** | 1894.88 **b** | 1860.96 **b** | 1591.97 **b** | 1796.63 **b** | 1329.34 **b** | 2018.34 **b** |
| 28 | 1852.63 **a** | 2124.72 **a** | 2131.78 **a** | 1820.06 **a** | 2117.65 **a** | 1732.94 **a** | 2215.24 **a** |
| 35 | 754.65 **f** | 961.08 **g** | 963.51 **e** | 869.15 **f** | 961.60 **e** | 753.61 **e** | 1084.88 **f** |
| 42 | 1159.35 **c** | 1395.24 **c** | 1302.92 **c** | 1171.09 **c** | 1274.63 **c** | 992.43 **d** | 1468.15 **c** |
| *p*-value(model) | <0.0001 | <0.0001 | <0.0001 | <0.0001 | <0.0001 | <0.0001 | <0.0001 |
| *p*-value(time) | <0.0001 | <0.0001 | <0.0001 | <0.0001 | <0.0001 | <0.0001 | <0.0001 |
| Microorganism | *Pediococcus parvulus* | | | | | | |
|  | Sensor Identity | | | | | | |
| Storage Days | AHS intensity | ANS intensity | CPS intensity | CTS intensity | NMS intensity | PKS intensity | SCS intensity |
| 0 | 867.51 **e** | 1294.65 **e** | 1075.30 **d** | 930.96 **e** | 1023.19 **d** | 1284.15 **c** | 1194.06 **d** |
| 7 | 631.02 **g** | 995.40 **f** | 882.70 **g** | 947.24 **d** | 688.49 **g** | 463.54 **g** | 921.26 **g** |
| 14 | 885.61 **d** | 1327.10 **d** | 1070.29 **e** | 708.23 **g** | 931.17 **f** | 584.16 **f** | 1181.33 **e** |
| 21 | 1709.81 **b** | 1894.90 **b** | 1862.38 **b** | 1587.20 **b** | 1807.64 **b** | 1353.65 **b** | 2015.27 **b** |
| 28 | 1817.05 **a** | 2096.68 **a** | 2099.69 **a** | 1790.88 **a** | 2095.95 **a** | 1684.42 **a** | 2181.31 **a** |
| 35 | 730.40 **f** | 944.03 **g** | 940.04 **f** | 846.46 **f** | 942.10 **e** | 718.91 **e** | 1061.26 **f** |
| 42 | 1180.78 **c** | 1410.64 **c** | 1319.75 **c** | 1193.80 **c** | 1289.46 **c** | 1015.35 **d** | 1488.74 **c** |
| *p*-value(model) | <0.0001 | <0.0001 | <0.0001 | <0.0001 | <0.0001 | <0.0001 | <0.0001 |
| *p*-value(time) | <0.0001 | <0.0001 | <0.0001 | <0.0001 | <0.0001 | <0.0001 | <0.0001 |
| Microorganism | *Lactobacillus brevis* | | | | | | |
|  | Sensor Identity | | | | | | |
| Storage Days | AHS intensity | PKS intensity | CTS intensity | NMS intensity | CPS intensity | ANS intensity | SCS intensity |
| 0 | 867.20 **e** | 1289.15 **c** | 942.25 **e** | 1067.75 **d** | 1065.51 **d** | 1247.55 **e** | 1198.13 **d** |
| 7 | 681.90 **g** | 560.67 **g** | 1045.07 **d** | 796.72 **g** | 938.08 **f** | 1062.56 **f** | 991.68 **f** |
| 14 | 887.54 **d** | 614.37 **f** | 704.88 **g** | 978.76 **f** | 1060.32 **d** | 1296.78 **d** | 1182.77 **d** |
| 21 | 1704.29 **b** | 1376.71 **b** | 1585.10 **b** | 1870.47 **b** | 1855.62 **b** | 1867.52 **b** | 2018.64 **b** |
| 28 | 1834.06 **a** | 1733.88 **a** | 1798.36 **a** | 2144.35 **a** | 2098.68 **a** | 2077.43 **a** | 2200.69 **a** |
| 35 | 745.46 **f** | 788.89 **e** | 866.48 **f** | 1001.54 **e** | 953.54 **e** | 929.54 **g** | 1090.63 **e** |
| 42 | 1186.94 **c** | 1065.92 **d** | 1194.77 **c** | 1342.44 **c** | 1327.19 **c** | 1394.10 **c** | 1515.33 **c** |
| *p*-value (model) | <0.0001 | <0.0001 | <0.0001 | <0.0001 | <0.0001 | <0.0001 | <0.0001 |
| *p*-value (time) | <0.0001 | <0.0001 | <0.0001 | <0.0001 | <0.0001 | <0.0001 | <0.0001 |
| Microorganism | *Wickerhamomyces anomalus* | | | | | | |
|  | Sensor Identity | | | | | | |
| Storage Days | AHS intensity | ANS intensity | CPS intensity | CTS intensity | NMS intensity | PKS intensity | SCS intensity |
| 0 | 875.71 **e** | 1190.23 **e** | 1078.97 **d** | 850.41 **f** | 1021.39 **d** | 1270.28 **c** | 1167.48 **e** |
| 7 | 679.06 **g** | 1112.10 **f** | 947.87 **g** | 1023.76 **d** | 734.86 **g** | 528.21 **g** | 985.06 **g** |
| 14 | 883.33 **d** | 1331.09 **d** | 1072.21 **e** | 709.35 **g** | 935.61 **f** | 574.19 **f** | 1182.57 **d** |
| 21 | 1712.11 **b** | 1896.42 **b** | 1864.52 **b** | 1592.52 **b** | 1804.76 **b** | 1356.19 **b** | 2018.65 **b** |
| 28 | 1809.35 **a** | 2088.77 **a** | 2090.16 **a** | 1782.06 **a** | 2080.53 **a** | 1670.21 **a** | 2173.40 **a** |
| 35 | 759.48 **f** | 969.08 **g** | 968.82 **f** | 871.37 **e** | 960.53 **e** | 757.80 **e** | 1089.62 **f** |
| 42 | 1152.44 **c** | 1389.85 **c** | 1295.37 **c** | 1166.65 **c** | 1266.79 **c** | 981.59 **d** | 1461.22 **c** |
| *p*-value(model) | <0.0001 | <0.0001 | <0.0001 | <0.0001 | <0.0001 | <0.0001 | <0.0001 |
| *p*-value(time) | <0.0001 | <0.0001 | <0.0001 | <0.0001 | <0.0001 | <0.0001 | <0.0001 |
